# Further Report of the Commissioners in Lunacy to the Lord Chancellor

**Published:** 1847-10-01

**Authors:** 


### Further Report of the Commissioners in Lunacy to the Lord Chancellor. Presented to both Houses of Parliament. 8vo. pp. 500. 1847.

The article on Insanity in our present Number was already in type when this "Report" came to hand; but so gratifying and interesting are some of its contents that we do not deem it advisable to postpone our notice of them. We have repeatedly had to bring under our readers' consideration the present imperfect provisions for the reception, management, and supervision of lunatics; and it is very gratifying to find that the Acts which recently received the sanction of the Legislature, have already done much to remedy some of these evils, and possess a sufficient power to cope with others yet remaining. We certainly were not prepared to anticipate that so much would have been accomplished in so short a time, and its having been so furnishes ample proof that the Commissioners have entered upon their task in good earnest, and are determined that their posts shall remain no sinecures. So far from this, a brief statement of the extent of the field over which their labours have to extend will show that these are very onerous, and the responsibilities incurred proportionally weighty.

Thus: in England and Wales alone there were at the beginning of the present year 177 County Asylums, Hospitals, and Licensed Houses; 437 separate establishments for single patients; and 596 Union Workhouses, containing imbecile or insane paupers, varying in numbers from 1 to about 100 in each workhouse. On the 1st Jan. 1847, these various establishments contained 26,516 lunatics: so that the aggregate number of these, together with the various committees, visitors, medical officers, attendants, and servants, cannot be fairly estimated at less than 30,000 persons. On a rough estimate, the aggregate amount of money annually expended for the maintenance of lunatics, or administered on their behalf, exceeds £750,000; and if to this amount be added the expense of maintaining many families cast upon the parish, and the interest of the large sums invested in the Public Lunatic Establishments, the total expenditure can be little less than 1,000,000 annually; while the capital invested in private and public establishments must amount to several millions. If to the 30,000 English lunatics above mentioned, we add the 12,397 returned as appertaining to Ireland, and the 3413 lunatic Scotch poor; together with the private patients, attendants, &c. in each country, we have an aggregate of nearly *fifty thousand persons* who are, in the United Kingdom, directly or indirectly, involved in the subject of Lunacy.

The Commission is thus composed: Lord Ashley (Chairman), Lord Seymour, Mr. Vernon Smith, Mr. Gordon, Mr. Barlow, Dr. Turner, Dr. Hume, Dr. Prichard, Mr. Procter, Mr. Mylne, Mr. Campbell, and Mr. Lutwidge (Secretary). Three of these gentlemen being physicians and three barristers, constitute the Visiting Commission, all visitations, according to the Act, being made conjointly by a physician and barrister. Ordinary meetings of the Commission are held at least once a week, at which the attendance of one legal and one medical Commissioner is required; while the most important subjects are referred to monthly meetings, at which all the Legal and Medical Commissioners, not otherwise urgently employed on the business of the Commission, attend. Respecting the business hitherto transacted, we find the following observations:—

"The necessity of visiting numerous Workhouses containing persons of unsound mind, has added exceedingly to the labours of the Visiting Commissioners. In effect, the whole time of the Legal and Medical Commissioners has been absorbed by the business of the Commission, by visitations; by examination of plans, estimates, and accounts; by forwarding the official correspondence; by long and frequent interviews with magistrates, architects, and private individuals; and by attending the Boards held for the despatch of business. Questions of considerable nicety have frequently occurred—some relating to the existing law, as it affects lunatics: others as it relates to County Asylums: others having reference to the state of mind of individual patients, the legality of their confinement, their fitness for liberation, their treatment whilst in confinement, and the due application of their property, &c.

"The amount of ordinary business transacted at this office has far exceeded our anticipations, and has rendered necessary the constant employment of several additional clerks. Besides which, the time and attention of the Board and of individual Commissioners have been much taken up by various inquiries of a special nature, which have considerably impeded the general business of the

Commission. In the case of the Haydock inquiries alone, the time of the four Commissioners was occupied (at two different periods) for 18 days; ten days at least, in addition, were devoted to the Reports consequent upon the inquiries; and eight meetings of the Board (each occupying upon an average six hours) were held, and entirely devoted to the purpose of receiving and considering evidence relating to this particular subject. Several other cases have also occurred which necessarily occupied considerable time, and required much deliberation.

"Without taking into consideration various meetings of Commissioners (not being regular Boards) or the daily attendance at the Office of the Legal and Medical Commissioners, when not engaged on Circuit, there have been held, in the course of the first 18 months of their Commission, 107 regular Board Meetings (our Chairman almost invariably presiding); in addition to which, the Legal and Medical Commissioners have each, upon an average, visited 409 Asylums and other places receiving Lunatics; have seen 17,749 patients; and have travelled 10,776 miles." P. 135.

Satisfactory is it to state that all this labour has not been in vain; and that, if in some points they call for amelioration, as a whole the new Acts work well and efficiently. Not only is supervision much more active and regular than heretofore; but it is more effectual, since the Commissioners can, through the medium of the Lord Chancellor or Secretary of State (and have done so), revoke the licences of private houses; and compel the adoption of improvements in those intended for paupers. To them, too, are referred the plans, estimates, and sites for the new asylums, in order that opinions resulting from their now extensive experience of the requirements of the Insane may be pronounced. Their interference, as the present Report exhibits, has been very beneficial in several instances. The result of the increased vigilance and power is, that a great improvement has been manifested in some of the establishments which, by the Report of 1844, were pronounced to be in a very faulty condition. The general disposition on the part of magistrates, proprietors, and others who have to do with the insane, seems to be the yielding a cheerful acquiescence in suggestions, which the Commissioners seem to exert a praiseworthy forbearance in not converting too hastily into peremptory orders; well knowing that, if adopted by the convictions of those concerned, they are far less likely to be evaded than if forced upon them. In extreme cases, however, they do not hesitate to exert their full and large powers. Many new county asylums are now in progress of erection; and the time allowed by the Act is rapidly approaching, when those of the magistracy who have neglected providing for their insane, will be compelled forthwith to do so. Then the disgraceful neglect of the insane, unavoidable under present circumstances stigmatised in the Report, will no longer exist; and we are pleased to find that the Commissioners are fully alive to the necessity of selecting the acute cases for prompt treatment, and providing due accommodation for those of a chronic character, which are now fast overwhelming and nullifying the advantages of present establishments. That these poor creatures will meet with all the attention and alleviation their cases admit of we feel persuaded; as we do that, with such an inspection and control as the Commissioners will be enabled to supply, the humane fears entertained by Dr. Conolly will prove groundless.

Of the improvement which has already taken place the Commissioners thus speak:—

"We are now desirous of satisfying your Lordship, as far as we are able, that, whatever defects may still be found in Lunatic Establishments, the amount of improvement that has taken place of late years, in the comforts and accommodations provided for the Insane, have been great and general. The Public Asylums have been in advance of the rest. The funds by which they are raised and supported, and the causes which influence those who have control over them, necessarily give them a superiority over private establishments. Indeed, we are fully convinced that the Lunatic poor will never be altogether properly provided for, until Public Asylums for the benefit of every county shall have been erected. At the same time we must observe, that there are some Private Asylums in which the pauper-patient is exceedingly well taken care of, and is as judiciously treated as in County Asylums; whilst, on the other hand, there are a few County Asylums which are inferior to many licensed houses.

"The improvement, as we have said, has been general in almost all the existing establishments. It is true that the progress has in no case, perhaps, been altogether regular and undeviating. Defects have frequently been observed; causes for animadversion have occasionally arisen; and every Establishment, however well conducted, has exhibited fluctuations, in respect to the improvement of the patients, easily accounted for, and depending upon various causes in no-wise affecting the character of the Institution. Thus the number of patients occupied varies with the season, a larger proportion than usual finding employment in those times when gardening and field-labour are especially required. The numbers subject to restraint sometimes exceed the average proportion, owing to the influx of new and violent cases, to the excessive heat or severity of the weather, the latter excluding them from exercise or recreation out of doors; whilst the number of recoveries will obviously depend on the number of recent cases received into the Asylum, or the bodily condition of the patients at the time of admission. Notwithstanding these and similar deductions, however, the improvement upon the whole is, in our opinion, undeniable." P. 64.

As the best evidence of the truth of the above statements, the Commissioners contrast the present state of the various Asylums, Hospitals, and Licensed Houses which have been stigmatized in the different Parliamentary and other Reports, with their former condition, which procured for them such unenviable notoriety. At the same time that they gratefully acknowledge the very great services and assistance rendered by that "most zealous, able, and intelligent body of men," the Medical Superintendents of the Public Asylums, and the ready co-operation of several of the medical officers and proprietors of Licensed Houses (the improvements often involving great outlays of money by these last)—yet the Commissioners properly state that to the *vigilant inspection of the insane* are the originating and maintaining improvements chiefly due, and that, if this is suffered to relax, no security whatever exists against even retrograde movements.

A portion of the Report is devoted to a brief account of the Special Investigations the Board has undertaken. Some of these, such as the Haydock Lodge Asylum, the illegal detention of a lunatic by a person calling himself Dr. Quail, and the prosecution of the attendants at Grove Hall, Bow, and the Nottingham Asylum, have been fully and recently noticed in the public prints; but to one or two other cases we may briefly advert. One of these, relating to the *arrest and imprisonment of a confirmed lunatic for debt,* filled us with astonishment and disgust, ignorant as we were, and we doubt most of our readers are, of the state of the law upon this point. In January last the Commissioners were informed that

Lieut. F., who had been for many years confined in Haslar Hospital, had been brought to Winchester Gaol under an arrest for debt, he being both insane and blind! Dr. Anderson, the Deputy-Inspector of Haslar, and the creditor, were summoned before the Board. Dr. Anderson stated that the unfortunate Lieutenant had inhabited Haslar for twenty years, during all which time he continued insane. He was entitled to a pension of five or six shillings *per diem* half-pay, from which one and sixpence were deducted, and the remainder paid over to his wife. By one of these violations of common sense which law seems privileged to commit, it seems that, although this poor creature was not responsible for his own actions, he was for those of his wife; and she having got into debt, he was arrested, the Lords of the Admiralty having commanded, under the advice of their solicitor, Dr. Anderson, to refrain from preventing process being served on him. The creditor who caused the imprisonment was a dissenting clergyman (alas!) and schoolmaster, to whom the wife had become indebted for the education of her children. Upon the strong remonstrance of the Commissioners this man consented to the liberation of his victim, after an incarceration of some weeks, which would, without such interposition, have been prolonged for three months more, during an inclement winter, until the arrival of the Insolvent Commissioners. After examining the state of the law upon the subject, the Commissioners observe—"It appears, therefore, that Lunatic Debtors are deprived of the benefits which the Law extends even to criminals;" and add—

"In reference to this case, we must observe, that attempts to proceed against lunatic patients are by no means infrequent, and that we have upon all occasions felt it our duty to interpose, as far as we are able, for the protection of the Insane. It appears to us that the *person* of a Lunatic should in every case be privileged from arrest and execution, and that some means should be taken (either by the appointment of a Guardian as Trustee or otherwise) to insure him sufficient means of defence to any suit or action that may be brought against him. Without some safeguard of this sort, any Lunatic Patient, however urgently he may require medical treatment, may be seized within the limits of an Asylum, and thrown, like Lieutenant F., into prison, to make good a debt for which he was never liable; and even where the person of a lunatic may not be taken, his property is liable to be distributed, for a debt to which he may have a valid legal defence." P. 150.

It is to be hoped that humanity and common-sense will not be again violated before an enactment embodying this suggestion can be passed. That such a one should still be wanting seems incredible.

The Asylum or Hospital for Lunatics at *Lincoln* is a strange place, and an account of an investigation of its condition entered into by the Commission is here given. It may be taken as proving by the *experimentum crucis* the utility of efficient and frequent inspection, for being a subscription asylum, it is *not* visited by the magistrates of the county, nor are its rules submitted to the Secretary of State, as is the case with County Asylums. The duties of inspection are self-performed by the body of persons who choose to constitute themselves Governors for Life by the payment of twenty guineas. And a despotic set they are, as may be judged of by the fact, that they pass decrees prohibiting their medical officers employing narcotics, and in various modes interfering with the other measures they may think fit to prescribe. There are three physicians attached to the place, each of whom successively takes the patients for a month. The same practice does or did prevail at the County Hospital: so that a patient might easily be prepared for an operation by one surgeon, undergo it at the hands of another, and have the after-treatment directed by a third! As in no town in the kingdom is the adage "Doctors differ" so completely verified as in this one, the patient has the full advantage or disadvantage of such a condition of things. The Governors seem to regard it as an excellent arrangement, and in their defence in answer to the criticisms of the Commissioners, observe: "The effect of the rotation is that of a standing consultation. The pointed attention of each Physician is drawn to the case by his individual responsibility (?); successful modes of treatment are adopted and continued; a course of unsuccessful treatment is discontinued; points of doubtful practice are forced into discussion." The physicians have the most diametrically opposite opinions upon the treatment of insanity; and it seems too bad to place the patient in the way of this cross-fire.

"One of them, Dr. Nicholson, advising classification; prescribing opiates occasionally to allay the restlessness of patients who are sleepless or in an excited state; and recommending the adoption of beer or wine as part of the ordinary diet, and frequently ordering them for particular patients; the other two (Dr. Charlesworth and Dr. Elmshirst) being adverse to classification; rejecting opiates in *all* cases; and ordering beer or wine very rarely, and then only as medicines in extreme cases when stimulants or tonics are imperatively required. It is worthy of remark that, there is a standing order of the Governors (who are *not* a medical body) by which the use of opiates to produce sleep, as also the use of beer and wine as parts of the diet, are prohibited. * * * * * * * It appears by the 'Physician's Journal,' that Dr. Nicholson visited the hospital throughout the whole of August, and that he was succeeded by Dr. Charlesworth, 1st Sept. Dr. Nicholson on the 30th Aug. directed porter to be given daily to 16 patients and wine to one. On the 1st Sept. Dr. Charlesworth ordered 12 of these to discontinue the porter, and the one to discontinue the wine." P. 359.

Other instances are added, but we need not cite them; the inherent mischievousness of such an arrangement being apparent enough. The Commissioners also object to the absence of due classification; to indecencies which have arisen from insufficient separation of the sexes: to the noise, turbulence, and frequent casualties; the allowing troops of strangers (311 in one month) to visit the wards; to the time of the House-surgeon being occupied by attending these idlers through the wards, and various other duties foreign to his proper province, which he is prevented fulfilling by an order of the Governors, that prohibits him from prescribing for the patients; to the insufficiency of the diet, &c. &c. &c. The Governors have made a most flippant reply, ill-suited to the gravity of the subject, and the courtesy with which the original strictures were delivered. We hope the time is not distant when an establishment possessed of such fine capabilities as this asylum, will not be permitted to waste them in the reckless manner it has hitherto done. That the non-restraint system ever succeeded under such a management speaks volumes for its power of universal application.

*Present System of Medical and Moral Treatment of Lunatics in the English Asylums.*—This forms the last part of the Report; and is unquestionably a document of great importance, as exhibiting the practice pursued at the chief asylums of this country, obtained by means of a circular letter containing queries addressed by the Commissioners to the various Medical Superintendents. To these, answers have been returned by upwards of fifty of these gentlemen, including in the number nearly every name of eminence in this department of medical science. The substance of these replies is analytically distributed for the purposes of reference, and some of them extend to a considerable length. We can here only indicate the general tenor of their statements, referring our readers to the Report itself, as containing the only authentic account of the opinions and practice prevailing among those whose opportunities constitute our authorities upon this important subject. The queries related to four principal topics: viz. the treatment adopted in Mania; in Epilepsy complicated with Insanity; in Paralysis connected with Insanity: and in Melancholia; and, upon consulting the various replies given at length in the Appendix, we find far less discrepancy of opinion than we were prepared for; and less we think than will be found to prevail upon most other medical topics.

1. *Treatment of Mania*—(1.) *General Bleeding.*—The replies are nearly unanimous in condemnation of this, and even the few physicians who approve of its employment under peculiar circumstances, just as strongly disapprove of it as an ordinary remedy in mania. The Drs. Fox of Brislington House make an important observation. "Previously to admission, most of our patients have been under medical treatment, and we have often had reason to suspect that the general bleeding to which they had been subjected has been detrimental, and that it has in some cases induced permanent fatuity;" and Drs. Miller and Shapter of St. Thomas's Hospital, Exeter, in like manner state that "the experience of its employment derived from those cases admitted after it had been freely practised, shows it to be evidently injurious, by breaking down the constitution, and conducing to an incontrollable mania, very apt to settle down into Dementia." Dr. A. Sutherland "considers the violent paroxysms of the acute stage as depending not on inflammation, but irritation. He thinks the arterial congestion which is found in cases p. m. the result not of inflammation but of irritation—an effort to repair the mischief sustained in some cases, and in others the effect of anæmia, which venesection would aggravate." Dr. Conolly considers it as "rarely admissible and generally dangerous in insanity." Those physicians who countenance it at all restrict its use to cases in which great plethora, threatening apoplexy, exists—never employing it as a mere means of quieting a paroxysm of excitement.

(2.) *Local Bleeding*, although condemned by some experienced practitioners, is, by the great majority, spoken well of.

(3.) *Emetics and Purgatives.*—The former of these, once so constantly employed in the treatment of mania, are now laid aside by nearly all experienced practitioners, the endeavouring to diminish excitement by smaller doses of antimony finding, however, favour in the eyes of several. "I am not in the habit of prescribing emetics," Dr. Sutherland observes, "for two reasons, 1, because in these cases, where there is a tendency to congestion in the capillary vessels of the head, they are known to increase it: 2, because the nerves of the stomach in insanity, as those of the intestines, are often less sensitive to impressions than in a state of health, owing to the disordered state of the functions of the brain, and it is sometimes necessary to give a large dose before the stomach will act." The beneficial effect of *Purgatives*, on the other hand, seems to be everywhere acknowledged, as indeed the frequency of the existence of constipation might have, *à priori*, led us to anticipate. Those of a strong character are generally preferred, and several practitioners speak very highly of the preferability of Croton oil.

(4.) *Anodynes.*—A prejudice long and generally prevailed against the use of this class of medicines in Insanity; but they are now looked upon by our most experienced physicians as very efficacious remedies in several forms of the disease—and especially so in cases of extreme violence and maniacal excitement. The different preparations of opium and henbane, but especially of the former, are those generally preferred: while, as to the efficacy of the Indian hemp, much difference of opinion seems to prevail, due, doubtless in some degree, to the difficulty of obtaining the substance genuine. Dr. A. Sutherland has some very interesting observations upon this class of remedies, a few of which we extract.

"These remedies are, according to my experience, of essential service in those cases of insanity which border closely upon delirium tremens, in cases of puerperal mania, in the acute stage, and particularly in the paroxysms and sleeplessness of mania, in cases where there is great nervous irritability from poverty of blood, and in cases combined with cachexia, from starvation and other causes. They seem to be contra-indicated when there are symptoms of incomplete general paralysis and congestion of the head. Prescribed merely because the case is one of insanity, without taking into consideration physical symptoms accompanying it, or not in proper doses, or not given sufficiently often during the day as well as during the night, these remedies disappoint the practitioner: they keep up irritation, and add to the excitement instead of allaying it. I have sometimes seen a very simple case converted into a very complicated one by the excessive use of anodynes. At St. Luke's I have been in the habit of prescribing the *Acetate of Morphia* in solution with distilled water—in private practice I often combine it with distilled vinegar (a very old remedy in insanity). The hydrochlorate is combined with advantage with dilute hydrochloric acid. I have found the meconiate of morphia very serviceable in cases where the two former preparations have not agreed with the patient. *Hyosciamus* and *Conium* are also very serviceable in the treatment of insanity. I am in the habit often of prescribing the former in those cases where it is essential that the bowels should not become constipated; and as it also acts upon the kidneys and skin, it is likewise useful when we wish the increase of the secretions of these organs. Combined with potassio-tartrate of Antimony, henbane is useful also in paroxysms of furor. I have seen considerable lassitude follow the administration of *træ. hyoscy.* ʒj. with ¼ gr. of the former ter die. This is, of course, in some cases, not to be desired. Combined with camphor, opium allays the irritability of those suffering under mania complicated with delirium tremens, and in the incipient paralysis of the insane, tartar emetic is the remedy I place most confidence in. *Conium* is very useful, either given alone, or in combination with hyosciamus and opium. The boasted effects of *Camphor* have not been realized to the extent, at least, which some of its advocates have insisted upon. I think, however, its effects in allaying uterine irritation cannot be doubted. The combination of hop, camphor, and henbane, is valuable in such cases. *Stramonium* is a remedy which has not succeded in my hands, although I have tried it in large doses." P. 391.

(5). *Baths* of various kinds, especially the tepid and shower, are well reported of in almost all the answers: many physicians have derived great advantage from the simultaneous application of cold to the head. Still, baths are seldomer resorted to in this country than is desirable; and far less often and for very much shorter periods than in France.

(6). *Diet and Stimuli.*—Medical practitioners connected with Asylums are nearly unanimous in recommending a generous diet, most of them advising the use of beer, wine, and other stimuli. As patients will oftentimes refuse food, Mr. Philipps observes they should at all times be well supplied with it, so that they may have access to it in the sudden fits and starts during which they will consent to take it. Dr. A. Sutherland frequently finds the various tonics of great use in the cases met with at St. Luke's, in some of which the insanity has indeed been induced by poverty and wretchedness.

2. *Treatment of Melancholia.*—There is, perhaps, less diversity of opinion as to the treatment of Melancholia than with respect to that of Mania. Most of the medical officers, who have given us an account of their practice in this form of mental disorder, seem to agree in directing their attention to the state of the alimentary canal, and the organs subservient to the digestive functions; and to be of opinion that, in cases of Melancholia the primary disease is often to be sought in some derangement there seated, and that great benefit may be derived from the means which tend to correct disorders of this class. Such are the use of purgatives, tonics, and stimulants of various descriptions. There is, however, likewise a prevalent opinion, expressed or implied, that the vascular system of the brain is in some manner oppressed and disordered in Melancholia either secondarily or primarily. Many of the remedies resorted to, seem to be prescribed upon this hypothesis, and if they are really efficacious and of benefit, which may be supposed from the fact that so many judicious and experienced persons agree and persevere in their use, this must be considered as affording evidence that the hypothesis in question is well founded.

"There is a greater amount of testimony for the beneficial effect of counter-irritation acting on the head in this disorder than in Mania; and even blood-letting, both general and topical, seems to have been in some instances fouud more useful in Melancholia than in maniacal affections. * * * * * * All the Medical Superintendents who have entered into the nature and purport of our enquiries, are unanimous in advising, as indispensable for the cure of melancholia, a regimen calculated to promote health and vigour of body and mind, viz., much exercise in the open air, cheerful society, abstracting the thoughts as much as possible from gloomy impressions, and some advise the use of wine and other stimulant drinks. Most are of opinion that if sleep does not follow a day spent in exercise of body, it should be procured by the use of some narcotic remedy, such as opium or henbane." P. 205-210.

Dr. A. Sutherland has known prolonged courses of mercury of great avail, and believes that medical treatment is frequently prematurely abandoned. Dr. Willis of Shillingthorpe considers the preliminary action of an emetic and drastic purge is essential; after which he has been in the habit of prescribing with great success the *tr. guiac. vol.* in *infus. cascarill.;* continuing to employ the warm-bath, exercise, and the flesh-brush, with occasional emetics, purgatives, and blisters.

3. " *Treatment of Epilepsy complicated with Insanity.*—Insane persons, subject to fits of Epilepsy, are generally supposed to be incurable; and there is reason to believe that, owing to the prevalence of such an impression, cases of Epilepsy, complicated with mental disorders, have been much neglected. We have not unfrequently seen, during our visits to Asylums, patients who had been brought from workhouses or from the cottages of their parents, where they have been many years subject to severe Epilepsy and reduced to a state bordering on fatuity, and who have been much improved in their mental and bodily condition after their admission into the Asylums. We have been informed that their paroxysms had become much diminished in frequency, and had in some instances ceased altogether, and that the mental faculties of these patients had become much less oppressed. This has been principally attributed by the medical officers to the improvement in diet, and the greater opportunities for exercise in the open air, inducing a general amelioration in the physical condition. That such instances would be more numerous than they are, if cases of Epilepsy were not neglected under the supposition of their hopeless nature, cannot be denied." P. 211.

When we consider the infrequency of cure in simple epilepsy, it is not surprising that an opinion of its incurability when complicating insanity should have become very general. Dr. A. Sutherland draws a distinction between epilepsy preceding and epilepsy succeeding insanity; and states his belief that the latter is *not* incurable, and may at least be alleviated. He prescribes, however, only the remedies ordinarily in use, and so frequently in vain, for epilepsy. Mr. Casson, of Hull, says he has never known a *chronic* case of epilepsy connected with insanity recover; and in all the patients whose brains have been examined by Sir A. Morrison at the Surrey Asylum, organic changes have been observed. Most practitioners seem to rely much on hygiene and diet as measures for mitigating the severity of the disease; but some difference of opinion prevails respecting the appropriate degree of fulness of diet allowable. "I have tried," says Dr. T. Prichard, "on a large scale, every remedy proposed in the works of various authors, upon the chronic cases that have come under my care, but in no one case successfully;" and Dr. Conolly states, "I have never seen a case of epilepsy in an adult permanently cured by any medicine whatever." We are disposed to think this is the true state of the matter; and although much may be done to improve the condition of these patients, and diminish the number of attacks, that they are never cured.

4. " *Treatment of the General Paralysis of the Insane.*—The peculiar form of disease distinguished by this name was not recognised and described till within a few years, though it must always have existed. It is now well known in all the large Asylums, as one of the principal causes of mortality among the male patients. It is most frequent among persons whose constitutions have been impaired by vicious courses and intemperance, and among those who have been reduced to extreme debility by want and other depressing causes. General paralysis has been almost invariably thought to be hopeless of recovery, and its victims usually perish within two or three years from the commencement of the disease." P. 217.

Dr. A. Sutherland, however, who seems more fortunate in the results of his practice than most of us, states he has met with three instances of recovery; and he seems chiefly to rely upon calomel and counter-irritation. This last, together with cold to the head and local bleeding, is employed by many others in the early stages of the disease. The Drs. Fox believe the disease may be sometimes arrested by the iodide of mercury, by prolonged open blistering of the scalp, and the use of the electro-galvanic apparatus.

5. *Moral Treatment.*—The Commissioners take a review of the present state of medical opinion upon this point; but having so recently adverted to the various topics it embraces, we will content ourselves with one extract.

"The principle of treatment adverted to in these remarks is important in two different points of view. In the first place, as furnishing a great part of the resources available for the cure of mental diseases, when the circumstances are such as to hold out a hope of recovery; and, secondly, as promising materially to lessen the sufferings and increase the comfort of incurable patients. In this last respect, it is of greater moment than any medicinal remedies. As a means of cure it ought never to be lost sight of. But there is reason to apprehend that the attention of medical men has been of late years too exclusively devoted to what is termed Moral Treatment, to the neglect, in some instances, of the resources of medicine. They appear occasionally to have lost sight of the fact, that insanity never exists without a physical cause, namely, some disturbance of the functions of the brain; disorders of the mind being only the result of some temporary or permanent derangement of the organism, by means of which all mental operations are carried on; whence it seems to follow that physical agents ought to be resorted to in the first instance, as the means of restoring the healthy and natural state. From the replies, indeed, which many of the Medical Officers of Asylums have given to the questions submitted to them by us, it may be perceived that the fact to which we have just adverted, has operated upon their minds, though there appears to be some variety in the methods in which they have acted under its suggestions. The conviction with which most of them seem to have been impressed is, that the disturbed state of the brain, which is the proximate cause of insanity in its various forms, is, in most instances, the result of disorder in some other part or function of the body, or of some serious derangement of the general state of health; and that the principal resources available for the cure of the cerebral affection consist in measures calculated to remove the original disorder of the physical or bodily functions, and to restore the health of the constitution in general. Hence the general recommendation of means likely to promote vigour of the body, such as exercise in the open air, ample diet, the careful administration of stimulants and tonics, bathing, warm clothing, and healthful recreations. Experience, as we might collect from the replies which have been received, if no other means of information existed, has fully confirmed the truth of this fundamental principle. It may, indeed, be observed that, in general, the number of recoveries from Insanity is found to be in proportion to the degrees in which the curative resources above alluded to have been employed. Under the old system of keeping patients bound hand and foot, in cells often dark, loathsome and disgusting, and feeding them with coarse and unwholesome diet, the result was an accumulation of chronic cases, and a frightful aggravation of human misery. The present humane method of treating the Insane, and the provision made, at the public cost, for Pauper Lunatics, of Asylums furnished with every resource for promoting health and comfort, exhibit in a striking point of view the intelligence of the age; and while they promise to diminish the numbers of the permanently insane, cannot fail to alleviate, in a great degree, the sufferings of that most afflicted class of human beings." P. 230.

